# Variability in Strength, Pain, and Disability Changes in Response to an Isolated Lumbar Extension Resistance Training Intervention in Participants with Chronic Low Back Pain

**DOI:** 10.3390/healthcare5040075

**Published:** 2017-10-16

**Authors:** James Steele, James Fisher, Stewart Bruce-Low, Dave Smith, Neil Osborne, Dave Newell

**Affiliations:** 1 School of Sport, Health and Social Science, Southampton Solent University, Southampton, Hampshire SO14 0YN, UK; james.fisher@solent.ac.uk (J.F.); stewart.bruce-low@outlook.com (S.B.-L.); 2 Department of Exercise and Sport Science, Manchester Metropolitan University, Manchester CW1 5DU, UK; D.d.smith@mmu.ac.uk; 3 Anglo European Chiropractic College, Bournemouth BH5 2DF, UK; NOsborne@aecc.ac.uk (N.O.); dnewell@aecc.ac.uk (D.N.)

**Keywords:** rehabilitation, deconditioning, individual response, heterogeneity

## Abstract

Strengthening the lumbar extensor musculature is a common recommendation for chronic low back pain (CLBP). Although reported as effective, variability in response in CLBP populations is not well investigated. This study investigated variability in responsiveness to isolated lumbar extension (ILEX) resistance training in CLBP participants by retrospective analysis of three previous randomized controlled trials. Data from 77 participants were available for the intervention arms (males = 43, females = 34) 37 participants data (males = 20, females = 17) from the control arms. Intervention participants had all undergone 12 weeks of ILEX resistance training and changes in ILEX strength, pain (visual analogue scale; VAS), and disability (Oswestry disability index; ODI) measured. True inter-individual (i.e., between participants) variability in response was examined through calculation of difference in the standard deviation of change scores for both control and intervention arms. Intervention participants were classified into responder status using *k*-means cluster analysis for ILEX strength changes and using minimal clinically important change cut-offs for VAS and ODI. Change in average ILEX strength ranged 7.6 Nm (1.9%) to 192.1 Nm (335.7%). Change in peak ILEX strength ranged −12.2 Nm (−17.5%) to 276.6 Nm (169.6%). Participants were classified for strength changes as low (*n* = 31), medium (*n* = 36), and high responders (*n* = 10). Change in VAS ranged 12.0 mm to −84.0 mm. Participants were classified for VAS changes as negative (*n* = 3), non-responders (*n* = 34), responders (*n* = 15), and high responders (*n* = 19). Change in ODI ranged 18 pts to −45 pts. Participants were classified for ODI changes as negative (*n* = 2), non-responders (*n* = 21), responders (*n* = 29), and high responders (*n* = 25). Considerable variation exists in response to ILEX resistance training in CLBP. Clinicians should be aware of this and future work should identify factors prognostic of successful outcomes.

## 1. Introduction

Chronic low back pain (CLBP) is one of the most prevalent medical disorders in today’s societies [1,2] representing an enormous economic cost worldwide [3,4,5,6]. Exercise is a common prescription for CLBP. This is despite the fact that previous Cochrane reviews have generally reported small effect sizes for most exercise approaches, reflecting either low average outcomes or high variability in outcomes [7,8]. But, these reviews have typically considered “exercise” as a single class of treatment without consideration to the varied exercise approaches that exist. The Cochrane reviews have not adequately described, defined and categorized the “exercise” studies they have examined and have been specifically criticized for this flaw and their wide-sweeping conclusions [7,8,9,10]. Though, this may be because many empirical studies of exercise in CLBP in fact lack an adequate description of the precise exercises used [11,12]. However, Searle et al. [13] recently examined the impact of different exercise types reporting that resistance training and motor control type exercises may offer the greatest benefits.

A general approach to exercise is further confounded by the fact that CLBP is a multifactorial condition [14,15]. Numerous models attempting to explain, predict, and integrate the multifactorial elements of CLBP have emerged within the literature [16,17,18]. Indeed, due to the multifactorial nature of CLBP, sub-grouping (i.e., splitting of the larger heterogeneous population of CLBP into smaller more homogenous groups) has been argued to aid in directing treatment [19,20,21].

Despite this, it has been suggested that specific deconditioning of the extensor muscles of the lumbar spine (lumbar extensor musculature i.e., thoracic and lumbar erector spinae, including the iliocostalis lumborum and longissimus thoracis, the multifidus, and also the quadratus lumborum when contracted bilaterally) may be a general risk factor for low back injury and pain [22,23,24]. Indeed, a recent review concluded that deconditioning of these muscles (reduced lumbar extension strength/endurance, atrophy, and excessive fatigability) is common in CLBP, and that this may be involved in many of the multifactorial symptoms and dysfunctions present [25].

As such, exercise designed to specifically condition this musculature (i.e., develop strength, endurance and hypertrophy [22,24,26,27] is often recommended. However, even within this category of exercise there exists a number of different approaches including: bench and roman chair trunk extensions (TEX), use of free weights (e.g., deadlifts, squats, good mornings), floor and stability ball exercise (e.g., TEX, bridging, 4-point kneeling), and resistance machines including those with and without restraints for isolated lumbar extension (ILEX) exercise [12]. Many of these approaches lack evidence for efficacy in conditioning the lumbar extensors; though resistance machines providing ILEX appear to be the exception [28]. A review of studies utilizing ILEX resistance training in CLBP suggests it produces statistically significant improvements in ILEX strength, pain, and disability, which also consistently meet minimal clinically important change values [29].

Resistance training in general, regardless of resistance type, commonly follows a general prescription [30,31] and often focuses on certain exercises to condition a specific muscle group such as ILEX [28]. A recent study comparing individualized low load motor control exercise with high load deadlift resistance training following a general approach reported no difference between groups for most outcomes in a homogenous population of participants with nociceptive mechanical CLBP [32]. An early study using ILEX resistance training also found that specific sub-grouping did not affect group outcomes, despite all participants receiving the same intervention [33]. Although, considering the heterogeneity of CLBP it might be expected that there would be at least some variability in the responsiveness of individuals to different treatments. Nelson et al. [33] also asked their participants to rate pain changes after an ILEX intervention on a 5-item scale (“worse”, “no change”, “slight decrease”, “decreased”, “substantially decreased”) reporting 64% rated a substantial decrease, 14% rated a decrease, 6% rated a slight decrease, 12% rated no change, and 3% rated a worsening of symptoms. Though in terms of average group outcomes exercise such as ILEX appears to be effective [29], the degree of variability in response in CLBP populations is not well investigated or understood.

Considering the paucity of data in this area it is of interest to investigate and further characterize the variability in responsiveness to ILEX treatment in CLBP participants. Such information is useful in helping consider characteristics which might influence response to training interventions in this population. The authors of the present piece have previously conducted a series of studies in which participants with CLBP have all undergone similar 12 weeks ILEX interventions, or corresponding control periods, and been examined for the same outcomes. Here the pooled data from these studies are reported with the aim of characterizing the variability in response of changes in ILEX strength, pain, and disability. It was hypothesized that there would likely be considerable *true* inter-individual (i.e., between participants) variation in responsiveness to ILEX resistance training in participants with CLBP due to the heterogeneity of the condition.

## 2. Materials and Methods

### 2.1. Experimental Approach to the Problem

The present study retrospectively examined data pooled from three previous trials of ILEX resistance training in participants with CLBP [34,35,36]. Participant data were extracted from the intervention arms of these studies that underwent 12 weeks ILEX interventions. In both Bruce-Low et al. [34] and Steele et al. [35] two manipulations of ILEX resistance training were examined (frequency and range of motion respectively), however, both studies found no difference between the two intervention arms of each trial. Thus all ILEX resistance training intervention and control arms of these trials were included. All participants in each trial had given their informed consent for inclusion before they participated in the study. Each study was conducted in accordance with the Declaration of Helsinki, and each study received individual ethical approval by the Ethics Committees of the lead author’s institution (11/40504/9).

#### Participants

A total of 77 participants data were available from the intervention arms (males = 43, females = 34) and a total of 37 participants data (males = 20, females = 17) from the control arms of the three combined trials. Participants had been recruited through posters, group emails, and word of mouth from the University and locality. In all trials participants had continued with any other treatments they were currently receiving, including medication, per recommendations from the reviewing ethics committee. Participants were however instructed to avoid beginning any other resistance training exercises designed to condition the lumbar extensor musculature. Inclusion criteria for each trial were as follows: participants who experienced nonspecific low back pain having lasted longer than 12 weeks and had no medical condition contraindicating resistance training. Exclusion criteria were any medical condition for which movement therapy might be contraindicated. These included acute (not reoccurring) low back injury occurring within the last 12 weeks, pregnancy, evidence of sciatic nerve root compression (sciatica), leg pain radiating to below the knee, paresthesia (tingling or numbness), current tension sign, lower limb motor deficit, current disc herniation, previous vertebral fractures, or other major structural abnormalities. In all trials participants were cleared to exercise by their general practitioner, physiotherapist, or chiropractor and provided written informed consent.

### 2.2. Procedures and Protocols

#### 2.2.1. Equipment

**Figure 1:**
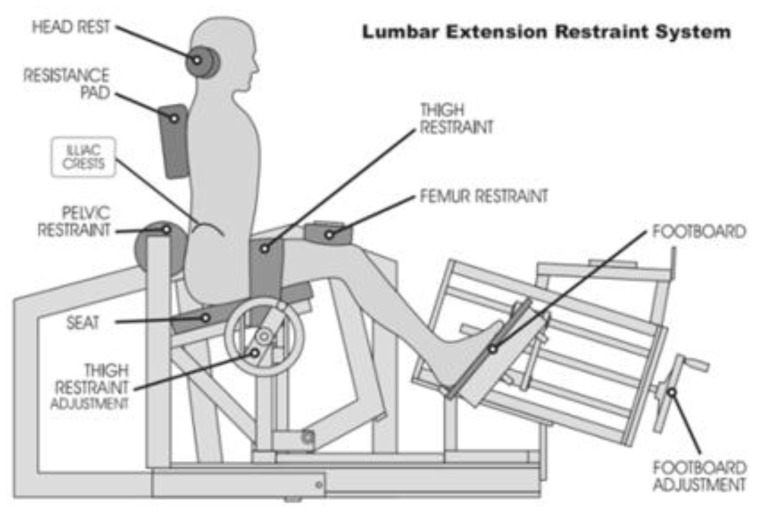
MedX Lumbar Extension Machine restraint system.

In all trials isometric strength testing and training for ILEX was performed using the MedX Lumbar Extension Machine (MedX, Ocala, FL, USA; Figure 1) which has been shown as reliable in both asymptomatic (*r* = 0.81–0.97 [37]) and symptomatic participants (*r* = 0.57–0.93 [38]) and is valid in measurement [39,40]. Pain was measured using a 100-mm visual analogue scale (VAS [41]) and disability measured using the revised Oswestry Disability Index (ODI [42]).

#### 2.2.2. Testing

In all trials isometric strength testing for ILEX was tested twice on separate days (at least 72 h apart to void residual fatigue or soreness) before and after the intervention. Each test involved maximal voluntary isometric contractions at various angles through the participant’s full range of motion. Briefly, after an initial light warmup and practice test at 50% of maximal perceived effort, participants performed isometric contractions where they increased effort gradually over a 3-second period until maximal. The restraint system was designed to prevent pelvic movement so that ILEX function could be tested independently. Details of the full-test protocol and its restraint system (Figure 1) have previously been documented elsewhere [37]. VAS (0 to 100 mm) and ODI (0 to 100 pts) were collected both before and after the intervention on the first and second to last visits to the laboratory where testing and training was conducted.

#### 2.2.3. Training for Intervention Arms

Training in all trials was conducted at a frequency of once [34,35,36] or, in the case of one of the training groups in Bruce-Low et al. [34], twice a week for 12 weeks. Participants performed a single set of variable resistance ILEX exercise through either a full [34,35,36] or limited (mid 50%) range of motion [35]. Resistance load was 80% of maximum isometric torque during baseline testing and participants performed repetitions until momentary concentric failure such that effort was controlled [43]. In the trial of Bruce-Low et al. [34] in the second weekly session participants performed a single set using 50% of their maximum isometric torque for 105–140 s and did not continue to momentary concentric failure. All repetitions were performed taking at least 2 s to complete the concentric phase, holding for 1 second in full extension, and taking at least 4 s to complete the eccentric phase. Resistance load in the set to momentary concentric failure was increased by 5% when participants were able to continue exercise for more than 105 s before reaching failure using their current load.

### 2.3. Statistical Analysis

Data for change as a result of the intervention (post minus pre) for the following outcomes were extracted from the trials: the average of ILEX strength across the angles tested, peak ILEX strength, ILEX strength index (calculated as the area under the curve of all angles tested using the trapezoidal method), VAS, and ODI. Normality of distribution was examined using the Shapiro-Wilk test. Variability within the cohort was examined for each outcome variable in several ways. Firstly, methods described by Atkinson and Batterham [44] were used to determine to examine whether true inter-individual variability in responses existed in the interventions arms independent of within-participant random variation. The difference in standard deviations of the changes in each outcome was calculated using the following equation:

$$\sqrt{\sigma i^{2}-\;\sigma c^{2}}$$

where *σi* is the standard deviation of the change scores for the intervention groups, and *σc* is the standard deviation of the change scores for the control groups. If the difference in standard deviations of the changes scores between the intervention and control groups is similar (i.e., close to zero) then no clinically important individual responses are evident. Where these were deemed to differ meaningfully from zero (considered as a difference in standard deviation favouring variability in the intervention arm approximately half of the mean change score), further analyses were conducted to attempt to categorise the nature of the individual responses in the intervention arms.

For each variable the coefficient of variation and each resultant distribution was described in terms of skewness and kurtosis. Data were pooled for each variable and rank ordered to identify the range for each outcome variable. From this bins were created at regular intervals and data were plotted as a histogram showing percentage of participants achieving the defined values for each bin. Histograms were produced using the data analysis toolbox in Microsoft Office Excel 2013 (Microsoft Corporation, Redmond, WA, USA). To further illustrate the distributions of the data Shapiro-Wilk testing, skewness and kurtosis was calculated using IBM SPSS Statistics for Windows (version 20; IBM Corp, Portsmouth, Hampshire, UK) and *p* ≤ 0.05 set as the limit for statistical significance. Lastly, responder status was also classified for outcome variables. For ILEX strength changes (both average, peak, and strength index) *k*-means cluster analysis was used to classify participants as either “low responders”, “medium responders”, or “high responders”. For VAS and ODI minimal clinically important change (MCIC) values as suggested by Ostelo et al. [45] were used to classify participants as either “negative responders” (increase in VAS or ODI), “non-responders” (reduction in VAS or ODI that did not meet MCIC), “responders” (reduction in VAS or ODI that met MCIC), or “high responders” (reduction in VAS or ODI that met double the MCIC).

## 3. Results

### 3.1. Participant Demographics

Demographic data were pooled from the included trials. Baseline demographics are presented in Table 1 for both control and intervention arms.

### 3.2. Presence of True Inter-Individual Responses

**Figure 2:**
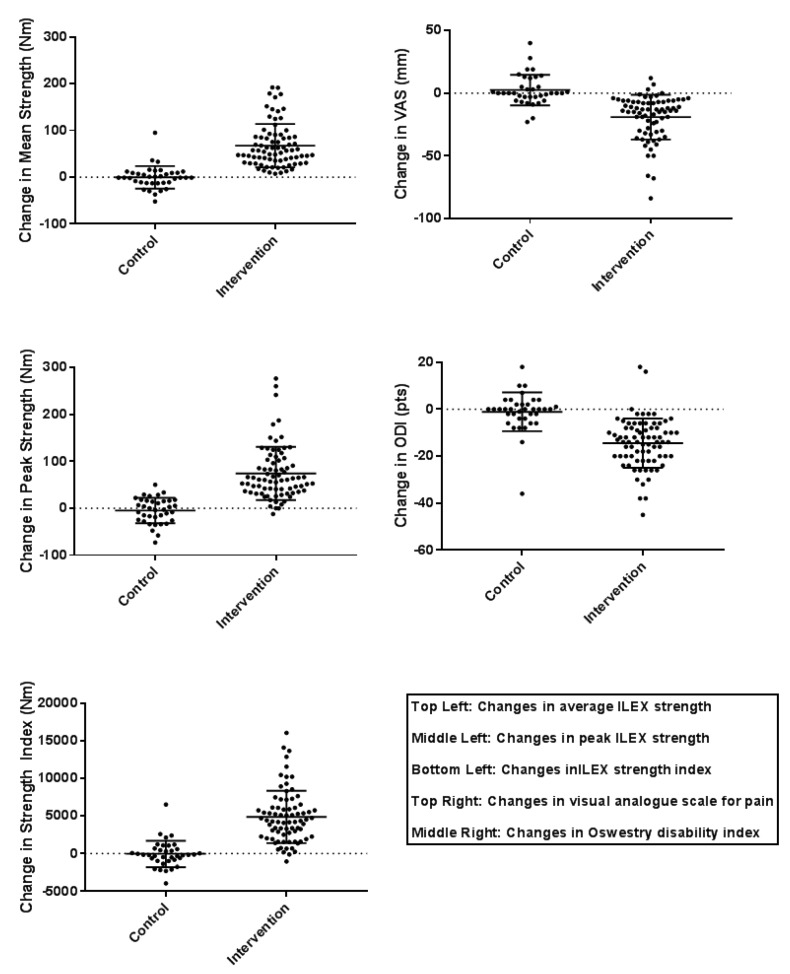
Individual responses.

All outcomes were considered to show evidence of true and meaningful inter-individual responses in the intervention arms (considered as a difference in standard deviation favouring variability in the intervention arm approximately half of the mean change score). Table 2 shows the calculated difference between the standard deviations of the change scores of intervention and control arms. Figure 2 shows the individual response plots along with the mean and standard deviations of the changes scores for each outcome in the control and intervention arms for visual comparison of the variation within each arm.

### 3.3. ILEX Strength

**Figure 3:**
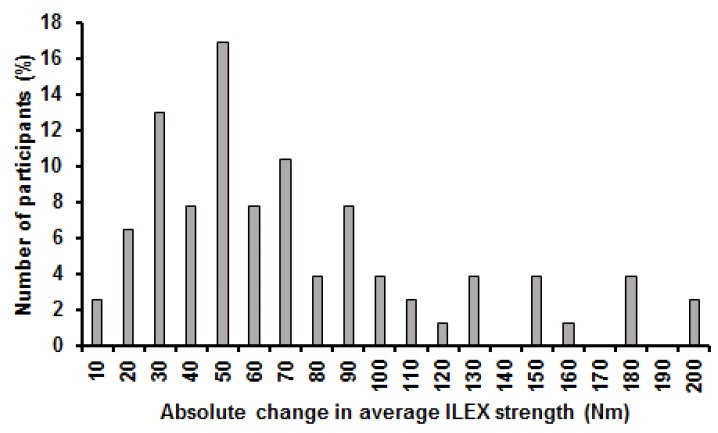
Histogram of changes in average ILEX strength.

**Figure 4:**
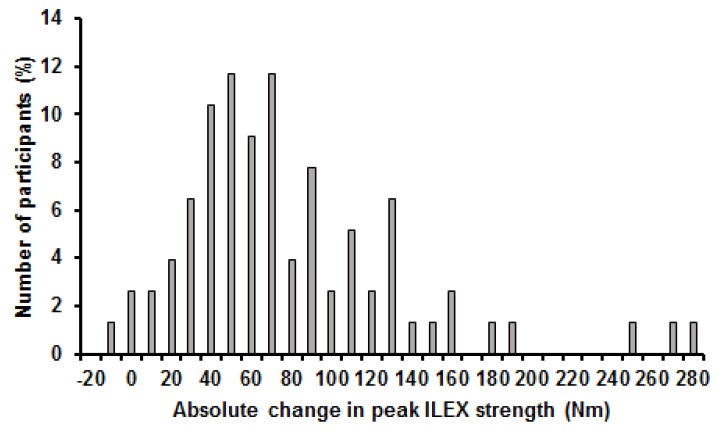
Histogram of changes in peak ILEX strength.

**Figure 5:**
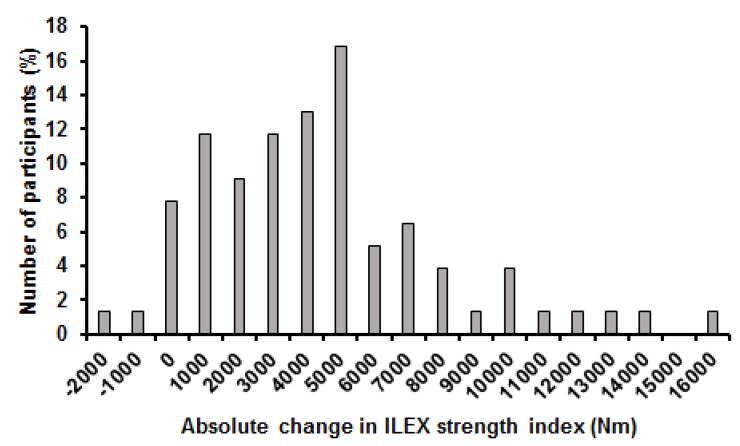
Histogram of changes in ILEX strength index.

Mean change in average ILEX strength was 67.5 Nm (42.5%) and ranged from 7.6 Nm (1.9%) to 192.1 Nm (335.7%). Change in average ILEX strength did not meet assumptions of normality of distribution when examined using the Shapiro-Wilk test (*p* ≤ 0.001). Coefficient of variation for change in average ILEX strength was 68.6% and the distribution showed a skewness of 1.1 and kurtosis of 0.6 (Figure 3). Mean change in peak ILEX strength was 74.2 Nm (29.2%) and ranged from −12.2 Nm (−17.5%) to 276.6 Nm (169.6%). Change in peak ILEX strength did not meet assumptions of normality of distribution when examined using the Shapiro-Wilk test (*p* ≤ 0.001). Coefficient of variation for change in peak ILEX strength was 76.5% and the distribution showed a skewness of 1.5 and kurtosis of 2.8 (Figure 4). Mean change in ILEX strength index was 4879.7 Nm (53.2%) and ranged from −1054.3 Nm (−17.5%) to 16,082.7 Nm (169.6%). Change in peak ILEX strength did not meet assumptions of normality of distribution when examined using the Shapiro-Wilk test (*p* = 0.001). Coefficient of variation for change in peak ILEX strength was 76.5% and the distribution showed a skewness of 1.0 and kurtosis of 1.2 (Figure 5).

Classification of responder status using *k*-means cluster analysis revealed distinct differences in both magnitude of ILEX strength change and number of cases between low (*n* = 31, mean change for average ILEX strength was 31.7 Nm, peak 44.3 Nm, and strength index 1846.9 Nm), medium (*n* = 36, mean change for average ILEX strength was 71.8 Nm, peak 74.1 Nm, and strength index 5582.7 Nm), and high responders (*n* = 10, mean change for average ILEX strength was 162.7 Nm, peak 167.7 Nm, and strength index 11,750.9 Nm).

### 3.4. Pain

**Figure 6:**
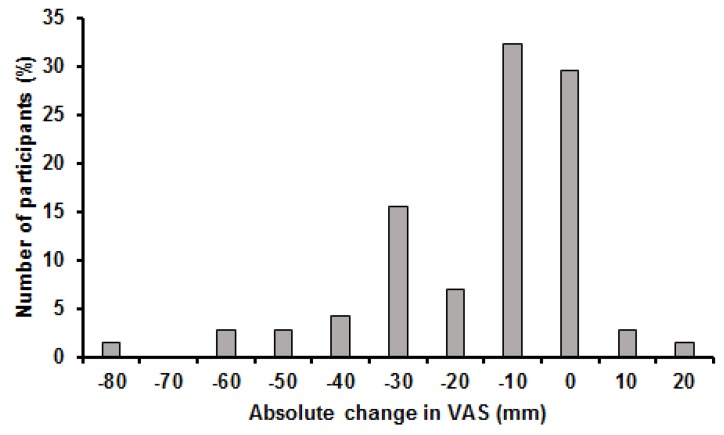
Histogram of changes in VAS.

Mean change in VAS was −19.1 mm and ranged from 12.0 mm to −84.0 mm. Change in VAS did not meet assumptions of normality of distribution when examined using the Shapiro-Wilk test (*p* ≤ 0.001). Coefficient of variation for change in VAS was 93.2% and the distribution showed a skewness of −1.3 and kurtosis of 2.2 (Figure 6).

Classification of responder status considering MCICs revealed distinct differences between numbers of cases in each classification for change in VAS; three participants classified as negative responders, 34 participants considered as non-responders, 15 participants considered as responders, and 19 participants considered as high responder (note: some data points were missing from original records for VAS in the study of Bruce-Low et al. [34]).

### 3.5. Disability

**Figure 7:**
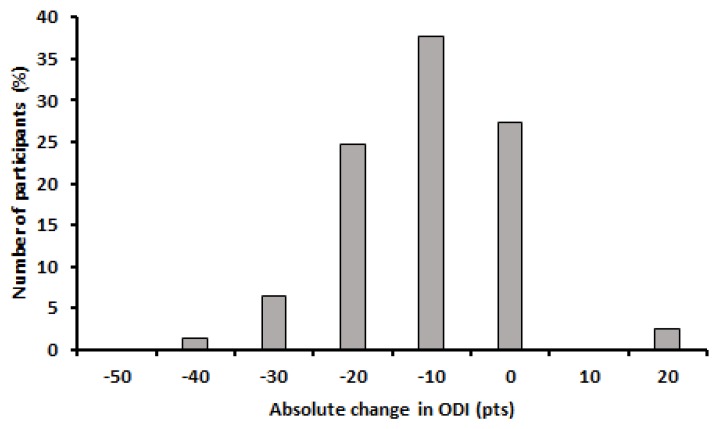
Histogram of changes in ODI.

Mean change in ODI was −14.4 pts and ranged from 18 pts to −45 pts. Change in ODI did not meet assumptions of normality of distribution when examined using the Shapiro-Wilk test (*p* = 0.049). Coefficient of variation for change in ODI was 72.4% and the distribution showed a skewness of 0.2 and kurtosis of 1.8 (Figure 7).

Classification of responder status using considering MCICs revealed distinct differences between numbers of cases in each classification for change in ODI; two participants classified as negative responders, 21 participants considered as non-responders, 29 participants considered as responders, and 25 participants considered as high responders.

## 4. Discussion

Although it appears well documented that exercise can produce beneficial outcomes in both pain and disability for people with CLBP [7,8,13], for which ILEX exercise seems particularly effective [29], the experiences of clinicians and theoretical models regarding the heterogeneity of CLBP suggest that some might benefit more than others. To date it appears that no study has attempted to quantify the variability in outcomes such as pain, disability, or functional outcomes such as strength, after controlled exercise interventions for CLBP. Indeed, though it is the experience of many, it has not previously been examined whether such variability in response reflects ‘true’ inter-individual responses or merely random within-participant variation. This study seems to be the first to document this in a moderately sized sample of participants with CLBP who had undergone 12 weeks of controlled ILEX resistance training based exercise. These results show there is true inter-individual variability in responses to an ILEX resistance training intervention with respect to strength, pain, and disability outcomes as evidenced by the meaningful differences between the standard deviation of changes scores between control and intervention arms in the studies examined. As such, the nature of this variability in outcomes was examined further.

ILEX based interventions are thought to work through the conditioning effect they have upon the lumbar extensor musculature [11,12,29]. This is thought to reverse the deconditioning of this musculature that may be present in those with CLBP [25]. As such, the variability in strength changes as a result of the ILEX intervention was examined here. Though not all participants showed an increase in peak ILEX strength or ILEX strength index as a result of the intervention there was an increase in average ILEX strength in all 77 participants examined. Despite this, the range of strength gains was wide in all strength outcome changes and reflects the considerable heterogeneity in response. It is well documented that strength gains after resistance training vary considerably amongst healthy individuals [46]. In terms of relative changes, Hubal et al. [46] reported a similar range of responses to those reported here, including some participants who showed decreases in isometric strength.

However, the coefficients of variation reported by Hubal et al. [46] are considerably lower (0.6% to 1.0%) than those reported here (68.6% to 76.5%). This may be reflective of the sample sizes in the respective studies; Hubal et al. [46] had 585 participants whereas the present study had 77. Alternatively, it may indicate that the variation in response to training is considerably higher in certain populations such as those with CLBP. Indeed, our cluster analysis revealed that the majority of participants (*n* = 50; 64%) could be considered as ‘low responders’ versus only a very small minority who were “high responders” (*n* = 5; 6%). Though their method of classifying responder status is not entirely clear, Hubal et al. [46] reported only 2.6% to 3.4% of their participants were considered low responders. It should be considered that most studies, including Hubal et al. [46], examining such variability in responses have not included a control arm in order to characterize “true” and meaningful response variation. Nevertheless, the differences in strength change variation between the study of Hubal and colleagues [46] and the present study may suggest that populations with CLBP may be inherently less responsive than asymptomatic populations in terms of strength gains.

This may be important as improved ILEX strength is thought to be the active mechanism through which improvements in pain and disability occur with ILEX resistance training interventions [11,12,29]. Indeed, some studies have reported an association between change in ILEX strength and improvement in pain [33,35]. Though, at least one has reported no significant association between these outcomes [47]. Thus whether or not a person’s ILEX strength improves or not as a result of an intervention may not be a good indicator of the success or failure of the intervention. However, it has been shown that ILEX strength is associated with outcomes such as lift capacity [48,49]. Thus, whether or not a person undergoing ILEX training has a substantial increase in ILEX strength may have implications for their wider functioning.

The reason for such variability and overall low response in strength is not clear from our results specifically. However, it may relate to fear avoidance beliefs. Al-Obaidi et al. [50] reported that fear avoidance beliefs were a prognostic factor for whether participants met MCICs for improvements in disability. It has also been shown that improvements in disability may be related to improvements in ILEX strength [35]. Participants undergoing the ILEX intervention in the studies included here were encouraged to train to momentary concentric failure (i.e., maximal effort); this due to the suggestion it might produce optimal strength and muscular adaptation [30,31]. However, it is possible that many may have not achieved momentary concentric failure, instead stopping short at a point they considered volitional failure due to fear avoidance beliefs. Recent studies have shown that stopping repetitions short of achieving momentary failure produces sub-optimal strength gains [51,52]. Further, a study examining ILEX resistance training in participants with CLBP where frequency, load, and effort where not controlled reported no change in multifidus cross sectional area [53]. Conversely, those which have had CLBP participants perform ILEX exercise to momentary failure have reported significant multifidus hypertrophy [54,55]. It is possible that a majority of participants did not train with a sufficient degree of effort to induce optimal muscular adaptations [56].

It is surprising that there is relatively little literature reporting variation in response to exercise based interventions for CLBP. Particularly as it is common for many practitioners to see patients who respond poorly or even negatively to treatments. Conversely, it is also known that some participants respond inexplicably well. Identifying whether such variation is indeed a result of inter-individual responsiveness and not merely random within-participant variation is of importance. Further, understanding the expected degree of variation in pain and disability outcomes as a result of exercise intervention might help guide practitioners to choose those which are more likely to produce favorable outcomes in the widest range of patients. The results presented here highlight there is considerable variation in responsiveness to ILEX resistance training for changes in pain and disability. This variation, in agreement with the strength outcomes, was similarly high (coefficients of variation were 93.2% and 72.3% for VAS and ODI respectively). Indeed, a considerable proportion of participants were either ‘negative responders’ or ‘non responders’ for both VAS (*n* = 37; 52%) and ODI (*n* = 23; 30%). However, in contrast to the strength data responder classifications, a large proportion of participants met MCICs and thus were considered as “responders” for VAS (*n* = 15; 21%) and ODI (*n* = 29; 37%). Further, a large proportion of participants achieved outcomes in excess of double the MCICs and were considered as ‘high responders’ for both VAS (*n* = 15; 27%) and ODI (*n* = 15; 33%). This would appear to support the experience of many practitioners as mentioned; some people respond inexplicably poorly or well to the same intervention.

These data would seem to agree with those reported by Nelson et al. [33] who found that a considerable proportion of participants reported a “substantial decrease” in their pain after an ILEX resistance training based intervention. As Nelson et al. [33] utilized a subjective global perceived outcome based scale it is difficult to compare their results directly to the classification here based upon MCICs. However, their range of responses appears similar. Despite there being a large proportion of persons who evidently respond well to ILEX interventions it is concerning to see a large proportion not achieving the MCICs, and also that some participants respond negatively. Why this is the case and whether or not these persons would do better with an alternative intervention is not clear. However, many argue that more positive outcomes may occur from interventions that are individualized based on sub grouping to the person receiving them [19,57]. As noted, some work has shown that psycho-social factors may play a prognostic role in determining whether persons will meet MCICs from exercise interventions, and specifically ILEX [50]. It is not yet clear whether this is a determinant of clinical improvement, or an effect of the intervention [56,58]. Yet, this does pose the question of whether or not characteristics can be identified that enable clinicians to predict success or failure with a particular intervention.

Thus far this concept currently has had little validating evidence to support it [59]. However, this may be due to inappropriate sub-grouping of participants in earlier studies [19,57]. The present study included participants broadly classified as having nonspecific CLBP with only certain red flag presentations excluded. As such, this sample likely represents the typical heterogeneity of CLBP and thus the true variation in responsiveness seen here may be a result of this.

The results presented reveal the considerable variation in response to ILEX resistance training in participants with CLBP. Range of variance in strength gains was similar to that seen in healthy persons and all participants increased in strength. However, the actual variance suggested that change in strength for persons with CLBP is more heterogeneous and that a considerable proportion of persons could be considered as “low responders” and only a very small proportion as “high responders”. Contrastingly, for pain and disability, though a significant proportion of participants did not meet MCICs and thus were considered “non responders”, a large proportion did meet MCICs, and a similarly large proportion exceeded these by more than double. From this it can be concluded that, though considering group average outcomes ILEX resistance training significantly improves pain and disability, some participants evidently respond far better than others. The reasons for this variation are not presently clear though may relate to the heterogeneity of symptoms experienced by persons with CLBP. It has been suggested that better methodologies for development and validation of classification tools are required to properly test the value of classification. This would include tandem utilization of both statistical and judgmental approaches to classification using a range of dimensions including patho-anatomical, symptomology, psychological and social. In addition to studies considering predictive validity of classification systems to produce desirable treatment responses [19]. This is certainly a route for future research and indeed trials have been proposed for this purpose [60,61,62,63]. Thus, the true variation in response identified here suggests that future work should consider this and look to identify characteristics that are prognostic of successful outcomes with ILEX resistance training. These might then help practitioners sub group and make better treatment decisions.

Limitations of the present investigation should be noted. For example, participants in the included studies were instructed to continue with any other treatments or therapies they were currently receiving but to avoid any new ones. Further, the specific nature of the symptoms (e.g., specific location) or details of the history of the participants CLBP occurrence (e.g., first occurrence), although considered in screening participants for exclusion criteria, were not documented for consideration in our analyses. This, in addition to other potential anatomical sources of pain, could thus not be considered and may have also influenced the variation in responses. As such, there could have been confounding variables in the present analysis. This was however the case for both intervention and control arms in all trials. Lastly, the sample size included here was relatively speaking small for the purposes of examining inter-individual responses. Such studies often include large sample sizes (e.g., *n* > 1000) [44]. The general lack of studies examining ILEX interventions means that there is less data available rendering difficulty in achieving this number of participants. However, small sample sizes are more likely to result in a negative difference in individual responses (i.e., one which favors greater inter-individual variation in the control arm) [44]. Our data instead consistently suggested greater variation in responses was present in the intervention arm.

## 5. Conclusions

A considerable proportion of persons with CLBP could be considered as ‘low responders’ and only a very small proportion as ‘high responders’ for changes in strength after ILEX resistance training. Contrastingly, for pain and disability, though a significant proportion of participants did not meet MCICs and thus were considered “non responders”, a large proportion did meet minimal clinically important changes and a similarly large proportion exceeded these by more than double. Though group average outcomes to ILEX resistance training appear favorable some participants evidently respond far better than others. The reasons for this variation are may relate to the heterogeneity of symptoms experienced by persons with CLBP. An important limitation in the studies examined was that participants were labelled as “non-specific”. Sub-grouping was not performed and may have implications for responsiveness to ILEX resistance training interventions. Thus, practitioners should expect that their clients might respond differently to interventions aimed at conditioning the lumbar extensor musculature and at present it might be most prudent to consider ongoing evaluation of pain and disability outcomes during an intervention to discern whether or not continuation is worthwhile. For example, if after a period of intervention a participant is not responding as desired then it may be worth considering changing to an alternative intervention approach, or stopping intervention entirely.

## Figures and Tables

**Table 1:** Participant baseline demographic data (Mean ± SD).

Variable	Control Arms (*n* = 37)	Intervention Arms (*n* = 77)
Age (years)	47 ± 14	46 ± 14
Stature (cm)	172.5 ± 10.0	171.3 ± 8.1
Body Mass (kg)	79.6 ± 16.4	77.2 ± 14.0
Body Mass Index (kg·m^2^)	26.5 ± 3.8	26.1 ± 3.2
Symptom duration (years)	14 ± 13	14 ± 13
VAS (mm)	26.9 ± 15.2	36.0 ± 21.6
ODI (pts)	30.0 ± 8.2	30.8 ± 12.8
Average ILEX Strength (Nm)	202.5 ± 99.7	198.9 ± 93.5
Peak ILEX Strength (Nm)	272.7 ± 135.0	276.1 ± 129.5
ILEX Strength Index (Nm)	13847.6 ± 6811.4	13078.0 ± 6780.9

VAS = visual analogue scale; ODI = Oswestry disability index; ILEX = isolated lumbar extension.

**Table 2:** Calculated differences between standard deviations (σ) of changes scores between control and intervention arms and comparison to mean intervention and control change scores.

Variable	Difference ($\sqrt{\sigma i^{2}-\;\sigma c^{2}}$)	Mean Intervention Change Score	Mean Control Change Score
VAS (mm)	13.0	−19.1	2.5
ODI (pts)	6.4	−14.4	−1.1
Average ILEX Strength (Nm)	39.5	67.5	−0.2
Peak ILEX Strength (Nm)	49.8	74.2	−4.7
ILEX Strength Index (Nm)	3022.4	4879.7	−37.2

VAS = visual analogue scale; ODI = Oswestry disability index; ILEX = isolated lumbar extension.
